# Quantification of cerebrospinal fluid flow in dogs by cardiac‐gated phase‐contrast magnetic resonance imaging

**DOI:** 10.1111/jvim.15932

**Published:** 2020-12-04

**Authors:** Muriel A. Christen, Daniela Schweizer‐Gorgas, Henning Richter, Fabiola B. Joerger, Matthias Dennler

**Affiliations:** ^1^ Division of Clinical Radiology, Vetsuisse Faculty University of Bern Bern Switzerland; ^2^ Clinic of Diagnostic Imaging, Vetsuisse Faculty University of Zurich Zurich Switzerland; ^3^ Department of Clinical Diagnostics and Services, Vetsuisse Faculty University of Zurich Zurich Switzerland

**Keywords:** baseline, canine, flow velocity, MRI

## Abstract

**Background:**

Cerebrospinal fluid (CSF) flow in disease has been investigated with two‐dimensional (2D) phase‐contrast magnetic resonance imaging (PC‐MRI) in humans. Despite similar diseases occurring in dogs, PC‐MRI is not routinely performed and CSF flow and its association with diseases is poorly understood.

**Objectives:**

To adapt 2D and four‐dimensional (4D) PC‐MRI to dogs and to apply them in a group of neurologically healthy dogs.

**Animals:**

Six adult Beagle dogs of a research colony.

**Methods:**

Prospective, experimental study. Sequences were first optimized on a phantom mimicking small CSF spaces and low velocity flow. Then, 4D PC‐MRI and 2D PC‐MRI at the level of the mesencephalic aqueduct, foramen magnum (FM), and cervical spine were performed.

**Results:**

CSF displayed a bidirectional flow pattern on 2D PC‐MRI at each location. Mean peak velocity (and range) in cm/s was 0.92 (0.51‐2.08) within the mesencephalic aqueduct, 1.84 (0.89‐2.73) and 1.17 (0.75‐1.8) in the ventral and dorsal subarachnoid space (SAS) at the FM, and 2.03 (range 1.1‐3.0) and 1.27 (range 0.96‐1.82) within the ventral and dorsal SAS of the cervical spine. With 4D PC‐MRI, flow velocities of >3 cm/s were visualized in the phantom, but no flow data were obtained in dogs.

**Conclusion:**

Peak flow velocities were measured with 2D PC‐MRI at all 3 locations and slower velocities were recorded in healthy Beagle dogs compared to humans. These values serve as baseline for future applications. The current technical settings did not allow measurement of CSF flow in Beagle dogs by 4D PC‐MRI.

**Abbreviations**
2D: two‐dimensional
4D: four‐dimensional
CKCS: Cavalier King Charles Spaniel
CSF: cerebrospinal fluid
FM: foramen magnum
FOV: field of view
NSA: number of signal averages
PC‐MRI: phase‐contrast magnetic resonance imaging
ROI: region of interest
SAS: subarachnoid space
SNR: signal‐to‐noise ratio
TR: repetition time

## 1 INTRODUCTION

The 3 main functions of cerebrospinal fluid (CSF) are the regulation of the intracranial volume, maintenance of the chemical environment of the central nervous system, and transport of biologically active substances. The choroid plexus as the sole source of CSF is debated, but its constant production creates a pressure that dictates the direction of the fluid flow through the ventricular system to the subarachnoid space (SAS).^1^, ^2^ This so‐called CSF bulk flow occurs over minutes. In addition, there is a pulsatile flow modulated by the expansion of intracranial vessels during every systole because of a mild increase in intracranial volume.^3^, ^4^, ^5^ As compensation, CSF is displaced from intracranial to spinal CSF spaces in systole and back in diastole, leading to a bidirectional pulsatile flow.^5^, ^6^, ^7^ Likewise, the respiratory cycle contributes to CSF flow—variations of intrathoracic pressure evoke changes in the epidural venous plexus.^8^ Abnormal CSF flow patterns and alteration of CSF flow velocity occur in a variety of diseases, mainly due to stenosis or obstruction of CSF spaces, which might result in secondary disorders of the ventricular system like hydrocephalus or cyst‐like lesions within the forebrain, spinal cord, or both.^9^, ^10^, ^11^, ^12^, ^13^, ^14^, ^15^, ^16^, ^17^, ^18^

Phase‐contrast magnetic resonance imaging (PC‐MRI) is a noninvasive method to quantify flow velocities and to characterize flow patterns. In two‐dimensional (2D) PC‐MRI, a bipolar gradient with equal magnitude induces a net phase shift of moving protons, which is proportional to their velocity. Cardiac gating encompasses bidirectional flow measurements during a cardiac cycle and velocity data are presented as coded grayscale values with negative values displayed as dark and positive values as bright pixels on the phase images, depending on flow direction and velocity.^19^, ^20^ New developments have enabled the measurement and visualization of flow in all 3 spatial dimensions and over time, known as four‐dimensional (4D) PC‐MRI.^21^, ^22^ In humans, PC‐MRI can determine the degree and location of CSF stenosis within the ventricles and the SAS, provide information for surgical planning, and assess postsurgical success.^7^, ^9^, ^10^, ^14^, ^16^, ^17^, ^23^

Toy breed dogs and brachycephalic dogs in particular show abnormalities of CSF spaces in MRI similar to humans.^24^, ^25^, ^26^, ^27^, ^28^ However, only limited velocity data are available in dogs and the only existing study in dogs demonstrated a lower peak velocity in Cavalier King Charles Spaniels (CKCS) compared to control dogs at the foramen magnum (FM). Furthermore, this investigation revealed that the combination of slower CSF peak velocity in the dorsal SAS at the level C2‐C3 intervertebral disc and higher peak velocity at the level of the FM in CKCS was associated with syringomyelia in CKCS.^29^ Despite these promising results, PC‐MRI is not implemented as a diagnostic tool to detect CSF flow abnormalities in dogs with hydrocephalus, syringomyelia, or other conditions indicative of stenosis of CSF flow. Challenges for the acquisition of PC‐MRI in dogs with sufficient signal to measure flow velocities arise from the smaller CSF spaces, higher heart rate in dogs, the slower flow velocity compared to humans,^29^, ^30^, ^31^ and the lack of normative data to compare with.

The aim of this prospective study is to optimize 2D and 4D PC‐MRI for the smaller CSF spaces and slower flow velocity in dogs, to provide normative values from neurologically healthy dogs with normal appearance of CSF spaces on MRI.

## 2 MATERIALS AND METHODS

### 2.1 Phantom study

The 2D and 4D PC‐MRI sequences were adapted for the anatomical differences in dogs compared to humans on a phantom mimicking the smaller CSF spaces and slower CSF flow velocity. A 20 × 15 × 15 cm plastic box was filled with gelatin, embedding 3 infusion lines with a diameter of 2.5 mm, 1.5 mm, and 1.0 mm as straight segments with a U‐turn. The infusion lines were connected to a medical perfusion pump outside the MRI room (Syramed μSP6000; arcomed, Regensdorf, Switzerland). The perfusion syringes were filled with water with a similar viscosity to CSF. The liquid flowed from the small to the large diameter infusion lines to reduce the effect of higher resistance in the small lines. Flow velocity was calculated with a linear stream‐profile conversion table (volume to flow velocity). Measurements were repeated when the velocity was gradually reduced.

The phantom was placed in a 3T magnetic resonance system (Philips Ingenia scanner, Philips AG, the Netherlands) with a SENSE‐head and neck coil. A cardiac frequency with a heartbeat of 100 bpm was simulated by a physiological simulation setting on the Philips software. 3D T1‐ and 3D T2‐weighted images were performed as planning sequences. The 2D and 4D PC‐MRI sequences were aligned perpendicular to the flow in the perfusion lines. First to be tested were sequence parameters for better signal‐to‐noise ratio (SNR) with a voxel size of 1 × 1 × 5 mm with different numbers of acquisitions (NSA 1 and 2), field of view (FOV 140/140, 150/150), and flip angle (15° and 20°). The measurements were performed once for each parameter and subjectively evaluated for good SNR. Finally, to find the closest velocity encoding the speed in the infusion lines, without getting aliasing but still having good SNR, different velocities encoding from 10 cm/s to 1 cm/s were tested. Additionally, for the 4D PC‐MRI sequence, both available accelerating techniques, kt‐BLAST and SENSE, were tested for their effect on scan time and signal.^19^, ^20^, ^32^

The image acquisition and assessment of image quality was performed by the first author, a PhD student with a Master's degree in veterinary medicine under supervision and advice of a senior radiologist (ECVDI Diplomate). Technical support concerning the 4D sequence was provided by a senior scientist in biomedical engineering with expertise in motion encoding.

Concerning the 2D sequences, further support was provided by a Professor of Radiology and Biomedical Engineering with a research focus on blood and CSF flow dynamics using flow‐sensitive MRI techniques.

A decision of sufficient image quality was made based on the velocity‐to‐noise ratio, that is, the ability to detect a change of gray scale value in time of a pixel undergoing a phase shift. The final decision on acquisition parameters was made in consensus between first and last authors.

### 2.2 Animals

**FIGURE 1:**
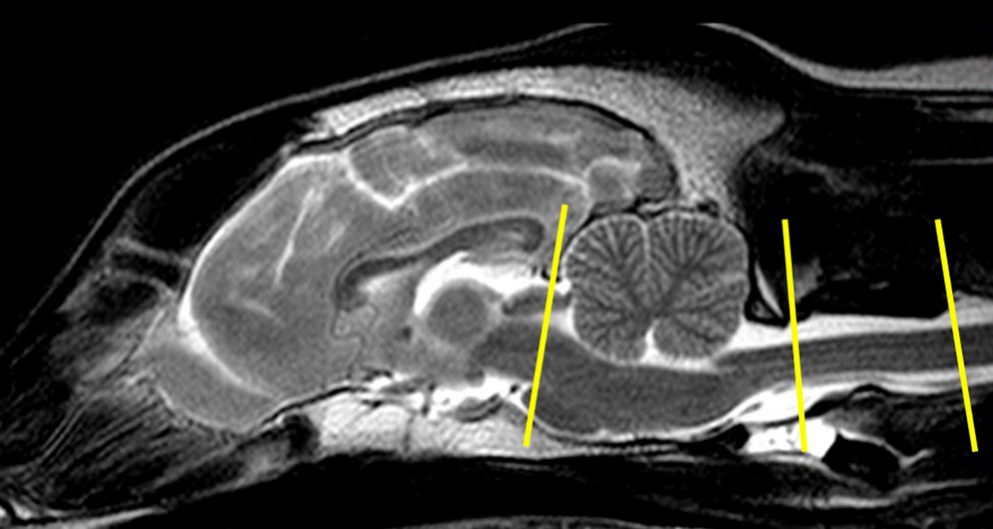
T2‐weighted mid‐sagittal magnetic resonance image of the brain and upper cervical spine of a Beagle. The yellow lines illustrated the location of the phase‐contrast sequence at the mesencephalic aqueduct, the most caudal aspect of the foramen magnum and at the atlantoaxial junction. Measurements were aligned perpendicular to the flow direction

The study was conducted with 6, adult, purpose‐bred, research Beagle dogs. Sex was equally distributed. The dogs were also used in another parallel research study, which did not interfere with our research aims and allowed the reduction of the number of experimental animals used (3R reduce). The study population had a mean age of 7.42 years (median = 7.65; range 5‐10) and a mean bodyweight of 13.6 kg (median 13.4; range 11.0‐18). All dogs were declared healthy on clinical neurological examination and showed no structural brain abnormalities on MRI (3D T2‐weighted and T1‐weighted images; Figure 1).

The dogs were fasted prior to anesthesia and premedicated with 0.2 mg/kg methadone (Methadon Streuli; Streuli Pharma AG, Switzerland) intramuscularly. An intravenous (IV) catheter (VasoVet 22G, Eickemeyer, Switzerland) was placed aseptically in the cephalic vein. Dogs were preoxygenated with a facemask with a flow of 4 L/min O_2_ for 5 minutes. Anesthesia was induced with propofol (Propofol 1% MCT Fresenius; Fresenius Kabi AG, Switzerland) titrated to effect. The tracheae of the dogs were intubated and sevoflurane (Sevorane, AbbVie AG, Switzerland) in O_2_ and air (Fi O_2_ = 60%) was administered to effect, depending on the plane of anesthesia, with an end‐tidal concentration between 1.8 and 3.3 vol%. The dogs were mechanically ventilated (Aestiva S5, 7900 SmartVent, Anandic Medical Systems AG, Switzerland) with a pressure‐controlled mode (8‐11 cm H_2_O). The respiratory rate was adjusted to achieve an end‐tidal CO_2_ of 35 to 42 mm Hg (4.66‐5.59 kPa). Acetated Ringer's solution (Ringer Acetat; Fresenius Kabi AG, Switzerland) 5 mL/kg/h) was administered during anesthesia. Body temperature was maintained between 37.5°C and 39°C and heart rate was maintained between 70 and 110 beats per minute. If cardiac frequency increased to over 110 bpm, a fentanyl (Fentanyl Sintetica; Sintetica, Switzerland) bolus of 2 μg/kg IV was administered and repeated, if necessary. Anesthesia monitoring included cardiovascular and respiratory variables, which were measured continuously and recorded by a multiparameter monitor (Datex S5, Anandic Medical Systems AG, Switzerland) that included pulsoxymetry, noninvasive blood pressure measurement, capnography, inhalant gas analysis, spirometry, and vectorcardiography. Mean blood pressure was kept above 65 mm Hg while heart rate was kept below 110 beats per minute. Anesthesia depth was adapted as necessary.

### 2.3 Magnetic resonance imaging

All dogs were scanned in sternal recumbency with the head slightly extended (<180° to the spinal cord) and elevated so that no pressure developed on the jugular veins.^3^, ^4^ The cardiac frequency was monitored by a magnetic resonance compatible peripheral pulse unit placed on the tongue and 2 magnetic resonance compatible electrocardiographic pads on each side of the thorax (Invivo 9312 ECG transmitter, Philips AG, Zurich, Switzerland). Plane of anesthesia and physiological parameters were stabilized if necessary with a fentanyl bolus before initiating CSF flow studies.

MRI acquisition was performed by the first author under supervision of a board certified senior radiologist. To exclude intracranial morphological abnormalities and for alignment of PC‐MRI sequences, 3D T1‐weighted (turbo field echo, repetition time [TR], 8.8 ms; echo time, 3.9 ms; flip angle, 8°; FOV, 13 × 136 × 150 mm; voxel size, 0.6 × 0.6 × 0.6 mm) and 3D T2‐weighted (turbo spin echo, TR, 2300 ms; echo time, 171 ms; flip angle, 90°; FOV, 170 × 130 × 157 mm; voxel size, 0.7 × 0.7 × 0.7 mm) sagittal sequence were acquired.

The first PC‐MRI sequence performed was a mid‐sagittal PC‐MRI sequence to visualize pulsation of the CSF flow and to identify possible obstructions of CSF flow. The parameters for CSF measurements in the sagittal plane were as follows: TR, 21 ms; echo time, 6.9; flip angle, 10°; FOV, 250 × 226 mm; slice thickness, 10 mm; voxel size, 0.98 × 1.4 mm; cardiac gating, retrospective; heart phases, 30; and velocity encoding, 5 cm/s. Velocity encoding was reduced if the signal was low or increased if aliasing occurred.

**FIGURE 2:**
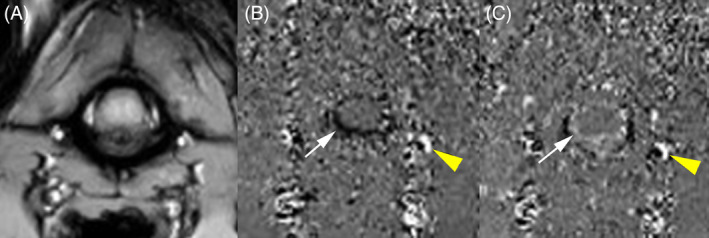
Magnitude (A) and phase images (B,C) at the level of atlantoaxial junction. On the phase image, every pixel carries velocity information. Cerebrospinal fuid (CSF) flow in rostrocaudal direction is displayed as negative velocity values and appears black (B, yellow arrow head). CSF flow in caudorostral direction is displayed as positive velocity values and appears white (C, yellow arrow head). Note the aliasing displayed as black and white pixels within the vertebral vessels (B,C: black arrow head)

Further 2D PC‐MRI sequences were acquired at 3 locations perpendicular to the flow direction (Figure 1). The first measurement was taken at the level of the mid‐ampulla of the mesencephalic aqueduct. The second measurement at the level of the caudal aspect of the occipital condyles to measure CSF within the SAS of the FM. The third location was the atlantoaxial joint. In 1 dog, an additional measurement was performed perpendicular to the SAS close to the intervertebral junction of C2/C3 after a low signal was detected on the phase images in the dorsal SAS of C1‐C2 in 2 dogs. The 4D PC‐MRI measurements covered the entire brain parenchyma and the cervical spine as caudal as C4 in a volumetric mode. The 4D measurements were performed once in each dog (Figure 2).

### 2.4 Postprocessing and image analysis

The postprocessing of the 2D data set was performed on an external workstation (MR WorkSpace 2.6.3.5, Philips Medical System, the Netherlands). The 4D PC data sets were processed by the GT‐flow software (Version 1.3.11, Gyrotools Ltd., Zurich, Switzerland).

**FIGURE 3:**
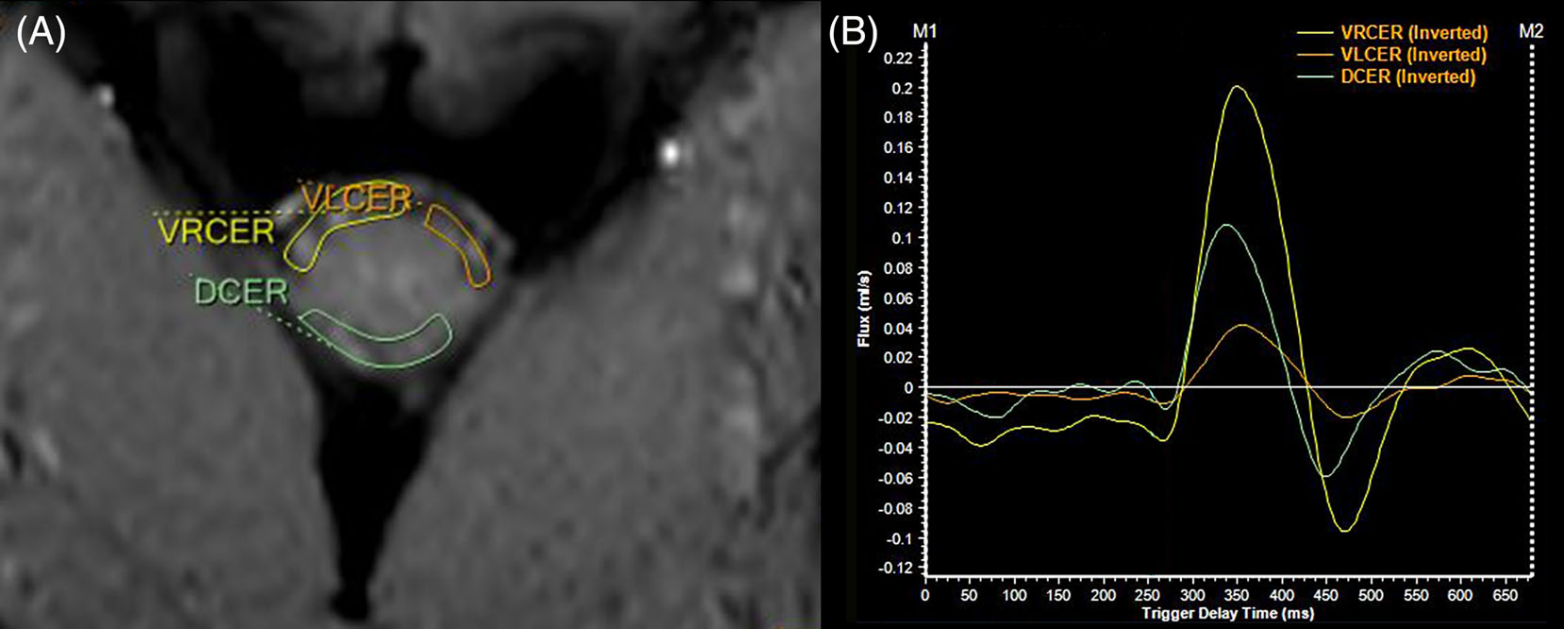
Transverse magnitude image close to the C2/C3 (dorsal is at the lower edge of the image) (A) with regions of interest (ROIs) drawn within the ventral (yellow and orange) and dorsal (green) subarachnoid space. Time velocity curves in B represent the flow (in mL/s over time in ms) of the corresponding ROI showing a biphasic flow pattern

Image analysis was performed by a single investigator (MC) who was instructed and supervised by a board certified senior radiologist. For all 3 locations, regions of interest (ROIs) were placed for each dog into CSF spaces using magnitude and phase‐contrast images in parallel in order to match information of flow with the anatomy. ROIs were propagated and manually adjusted to each of the 32 resulting phase images. One ROI was drawn at the level of the mid‐ampulla of the mesencephalic aqueduct. On images of the caudal aspect of the occipital condyles and the cranial cervical spine, 3 ROIs were placed in the SAS surrounding the spinal cord: 1 ROI in the dorsal SAS and 2 ROIs in the ventral SAS to avoid areas where no flow signal was present and to exclude the basilar artery or spinal artery from the velocity measurements (Figure 3).

The software generated automatically a time velocity curve shown as graph and listed mean, maximum, minimum, peak velocity in cm/s, and flux in ml/s. Results are reported in peak velocities (cm/s) since average flow underestimates velocity measurements.^4^, ^29^, ^33^

On the acquired data from the 6 dogs, all ROIs were placed 3 times at least 3 days apart and the mean of the peak velocity is reported.

Postprocessing of the 4D data set could not be performed due to high velocity noise and low SNR on the phase images, which led to undetectable velocity in CSF spaces.

### 2.5 Statistical analysis

Descriptive analysis was performed with all values obtained. The data were expressed as mean ± SD or median with range.

Intraobserver variability was assessed by calculating the SEM based on 3 measurements on each location of the 2D phase images. The statistical distributions of the velocities were visually examined using histograms and quantile plots and tested for normality using the Shapiro‐Wilk test (*P* ≤ .05, R 1.0.143 2009‐2016).

## 3 RESULTS

### 3.1 Phantom study

Best SNR was achieved using the following sequence parameters: TR, shortest (calculated by the scanner according to the VENC); echo time, shortest (calculated by the scanner according to the VENC); flip angle, 15°; FOV, 150 x 150 mm; slice thickness, 5 mm; voxel size, 0.58 × 0.83 mm; NSA, 1; heart phases, 32; cardiac gating, retrospective. Sequence acquisition time was no longer than 3 to 4 minutes. With these parameter settings, flow was visible on the phase images in all infusion line diameters (2.5‐1 mm) with velocities under 1 cm/s. In the phantom test, the calculation of the theoretical estimated velocities in tubes of different diameter corresponded with the measured 2D PC‐MRI velocities. Sufficient velocity‐to‐noise ratio for the volumetric measurements with the 4D PC‐MRI sequence was achieved using the following parameters: TR, shortest; echo time, shortest; flip angle, 5°; FOV, 140 × 140 × 40 mm; voxel size, 1 × 0.99 × 1 mm; stacks, 1; slices, 40; slice orientation, coronal; NSA, 1; heart phases, 16; cardiac gating, retrospective, and acceleration technique SENSE. Velocity encoding was selected at 10 cm/s in all spatial directions (RL‐AP‐FH). Alteration of these parameters to improve velocity‐to‐noise ratio and SNR, such as reduction of voxel sizes for detection of flow in small spaces or lowering velocity encoding, increased the time of the sequence to >14 minutes despite the use of SENSE acceleration. Flow signal could not be obtained on the phase images with acceleration of kt‐BLAST. A flow rate of >3 cm/s was visible with a low SNR within the 2 larger infusion lines (2.5 mm and 1.5 mm) but could not be detected in the smallest line (1 mm). Flow rates below 3 cm/s could not be measured with the 4D PC‐MRI sequence.

### 3.2 Animals

The 2D PC‐MRI imaging enabled the assessment of CSF flow in every dog. The acquisition time of CSF flow measurements at 3 locations was between 3 and 4 minutes, and heart rate was between 70 and 110 bpm. A bidirectional flow pattern was visible on the sagittal phase images in the dorsal and ventral SAS from the level of the FM to the cervical spine, but not cranial to it. On transverse sequences, a bidirectional flow pattern was visible in all ROIs except for the dorsal SAS of C1‐C2 of 2 dogs, where no signal could be detected. Velocity encoding for measurements at the level of the FM and the cervical spine was set at 5 cm/s for all dogs and then reduced to 3 cm/s at the level of the aqueduct for better SNR. The shortest TR and TE were calculated by the scanner according to the velocity encoding and was for all transverse 2D PC‐MRI sequences 16 to 17 ms and 10 ms, respectively. The acquisition time for T1 weighted, T2 weighted, and 2D PC‐MRI was 32 minutes; the 4D PC‐MRI sequences took additional 14 minutes.

The CSF peak velocities resulting from all 3 measurements among all dogs were normally distributed (Shapiro‐Wilk test *P* ≤ .05). The mean peak velocity of all 6 dogs for the different locations is listed in Table 1. Peak velocity was the lowest at the level of the aqueduct and the highest in the ventral SAS at the cervical spine. The peak velocities in the dorsal SAS of the FM and cervical spine were lower when compared to the ventral SAS. The peak velocities additionally measured at the level of C2/C3 in dog Nr. 6 were as follows: ventral right SAS 2.94 cm/s, ventral left SAS 2.62 cm/s, and dorsal SAS 1.46 cm/s. Peak velocities in the ventral SAS are higher at every measured position than in the dorsal SAS, with the exception of 1 dog. In this dog, a similar peak velocity at the level of FM was measured.

**TABLE 1:** Mean peak velocity in cm/s and SD of all 6 dogs for the different locations

		Foramen magnum			Cervical spine		
	Mesencephalic aqueduct	Ventral right	Ventral left	Dorsal	Ventral right	Ventral left	Dorsal
Mean peak velocity in cm/s	0.92 ± 0.5	1.78 ± 0.7	1.9 ± 0.8	1.17 ± 0.4	2.15 ± 0.8	1.91 ± 0.5	1.27 ± 0.4

Intraobserver variability for the manual placement and adjustment of the ROI on the 2D phase images was ≤0.68 cm/s for the SAS of FM and cervical spine and ≤0.13 cm/s at the level of the mesencephalic aqueduct. The 4D PC‐MRI images showed marked velocity noise, to the point that CSF spaces were not detectable anymore. Hence, CSF flow velocity measurements and visualization of flow were not possible with the 4D PC‐MRI sequence in any of the dogs.

## 4 DISCUSSION

The present study provides normative data of CSF flow in a group of middle‐aged neurological healthy dogs as baseline for comparison in future studies. Ventricular and subarachnoid CSF space abnormalities are common findings in dogs; however, their relationship to alterations of CSF flow is not well understood and poorly investigated.

PC‐MRI is routinely used as a method to quantify CSF flow in humans but needs adaptation for use in veterinary patients, because of the small CSF spaces,^30^, ^31^ slow CSF flow velocities, and higher heart rate compared to humans.^29^ The small CSF spaces require smaller voxel sizes that lead to a reduced SNR. Compensation can be achieved by an increased FOV or increased NSA, which produces prolongation of scan time.^19^, ^20^, ^34^ The peak velocities in dogs were approximately 20% lower compared to humans.^9^, ^15^, ^16^, ^21^, ^29^, ^35^ Lower flow velocities lead to higher velocity noise and require lowering of the velocity encoding. Reducing the velocity encoding results in an increased TR, which is accompanied by increased scan time.^19^, ^20^, ^22^, ^34^ Finally, PC‐MRI is cardiac gated, meaning that a cardiac gradient is applied in synchronization with the R‐R interval, the time elapsed between 2 successive R waves of the QRS signal on the electrocardiogram. A higher heart rate leads consequently to a smaller R‐R interval and therefore a shorter time to collect flow data and might result in longer acquisition time or even abortion of the sequence.^19^, ^20^ The phantom was built to mimic the conditions in veterinary patients, and sequence parameters were adapted until a satisfactory SNR within a clinically reasonable acquisition time of 3 to 4 minutes was achieved. The sequence parameters developed on the phantom were then applied in dogs, demonstrating that the phantom allowed us to optimize the 2D PC‐MRI sequence without having a dog in the scanner under general anesthesia. This is in line with 3R requirements and demonstrates that phantom studies in MRI could help to refine protocols before use on living animals and consequently reduce anesthesia time and risk. Furthermore, the use of the phantom allowed us to demonstrate that 2D PC‐MRI provides data corresponding to the calculated flow velocities in tubes. In contrast to 2D PC‐MRI where the velocity can only be decoded in 1 spatial direction, 4D PC‐MRI uses 3 bipolar gradients delivering time‐resolved CSF flow data in 3 dimensions. This allows the user to determine the exact location of measurement and flow direction in a postprocessing step.^21^, ^22^, ^36^ Despite using different acceleration techniques such as kt‐BLAST and SENSE,^22^, ^32^ an adaptation of velocity encoding <10 cm/s led to scan times of >20 minutes, which were considered as unacceptable for clinical use. Therefore, velocity encoding was kept at 10 cm/s, which resulted in increased velocity noise to such an extent that no measurements could be obtained. Current developments and advances of acceleration techniques might allow overcoming the challenges of small CSF spaces and low velocity within reasonable scan times in the future.

Therefore, the results presented here are limited to 2D PC‐MRI with CSF flow velocities measured at 3 different locations. These locations play an important role in the pathomechanism of common disease processes such as stenosis, hydrocephalus and syringomyelia.^9^, ^10^, ^11^, ^13^, ^15^, ^16^, ^17^, ^18^ In addition, measurements at different locations within 1 individual are important, as differences in peak velocity indicate stenosis.^29^ The measurement at the cranial cervical spine might provide additional information about intracranial compliance.^15^

At each of the 3 locations, the peak velocities measured in the study population of middle aged healthy Beagle dogs were lower than those measured in humans. This is especially true for the level of the mesencephalic aqueduct, where values ranging from 3 to 7 cm/s were obtained in healthy human participants.^9^, ^14^, ^37^, ^38^ The reason for the slower flow might be species‐related differences such as skull shape, the orientation of the brain to the spinal cord, or might be due to the smaller size of dogs compared to humans. Age of the individual could also play a role since a study comparing CSF flow velocity in human infants found a higher CSF flow velocity than that in adults.^33^ To reduce individual differences in CSF flow velocities, we only included dogs of the same breed and similar age and therefore similar conformation (size, bodyweight, and skull shape). These inclusion criteria resulted in a small sample size of 6 individual dogs, and the peak velocities measured at each of the 3 locations showed only small differences between dogs resulting in a small standard variation. Our results obtained from this small but consistent study sample will enable future comparison with measurements from larger samples with individuals of different ages, body weight, or skull shape. This will allow investigation of factors associated with CSF flow velocities in dogs. Apart from differences between individual animals, other factors influence the PC‐MRI measurements of CSF flow. First, variation might arise from the site of measurements, particularly at the level of the mesencephalic aqueduct, where normal velocities in humans encompass a broad range from 3 to 7 cm/s. This is probably caused by setting the phase encoding gradient at either the rostral part, the ampulla, or the caudal aspect.^5^, ^10^, ^17^, ^38^, ^39^, ^40^ The obtained range of 0.51 to 2.08 cm/s in the present study group for the peak velocity at the level of the aqueduct therefore seems acceptable and was obtained by defining landmarks during acquisition. Second, variation might arise from manually placing the ROI in the postprocessing step. Therefore, the use of semiautomatic software for ROI placement has been suggested.^41^ The software was not available for us, and ROI placement was performed 3 times with low intraobserver variability of 0.68 for all SAS spaces and below 0.13 within the mesencephalic aqueduct. In the present study, manual ROI placement was a low source of error, but variability between different observers might be higher. Well‐defined landmarks for both, measurements and ROI placement, seem to be of high importance to be able to compare velocities. In a previous study, CSF peak velocities of 59 CKCS dogs were compared to 5 controls, 4 Beagle dogs, and 1 mixed‐breed dog. The flow velocities measured at the FM and mean peak velocity in the ventral and dorsal SAS of the FM in the control group was 0.75 ± 0.24 cm/s and 0.59 ± 0.13 cm/s, respectively.^29^ The obtained peak velocities of the control group were lower compared to our study group and velocities obtained in CKCS were even lower.^29^ Reasons for the higher velocities found in the present study might be due to patient‐related factors such as the individual size of CSF space, jugular venous flow, and compliance of the intracranial space ^3^, ^4^, ^5^, ^15^ as well as the heart rate and respiration rate of the patient,^8^, ^33^, ^39^, ^40^ all factors known to influence the CSF flow velocity. A difference in the examination protocol might have influenced the measurements, all dogs in the previous study were positioned with the head in a flexed position. In our study, to facilitate comparison with future measurements, the dogs were examined with the head in an extended position, according to our institutional protocol for brain MRI. However, the extended head position in sternal recumbency resulted in a narrowing of the dorsal SAS, so that in 2 dogs no CSF flow data from the phase images could be extracted. It seemed that the dorsal SAS at the level of the atlantoaxial junction is especially narrow due to kinking and the dens axis, which displaced the spinal cord slightly dorsally.^30^, ^31^ For this reason, we performed an additional measurement at the level close to the intervertebral disc space of C2/C3 in 1 of the 6 dogs. This measurement resulted in a good signal of CSF flow in phase images. Therefore, cervical CSF flow measurements close to the intervertebral junction C2/C3 with well visible SAS spaces might be more reliable and clinically useful rather than the atlantoaxial junction. Furthermore, examinations in dorsal recumbency might facilitate visualization of the dorsal SAS, but the influence of this nonphysiologic position in dogs on visualization and resulting flow parameters is yet unknown.

The anesthesia protocol for this study was chosen because of the relatively low effects on the cardiovascular system. Of note, inhalational anesthetic agents could have a cerebral vasodilating effect and might increase cerebral blood volume and hence intracranial pressure, if the systemic blood pressure is below 60 mm Hg.^42^ To avoid differences related to physiological parameters, a standardized anesthesia protocol, monitoring and consistent conditions are important. Measurements performed under constant heart rate seem important to be able to detect differences in flow velocities at different locations in 1 animal. The obtained flow velocities in our dogs suggest that if at 1 location the flow velocity is lower (see Table 1, dog 5), the velocities at the other locations are lower too, and vice versa (see Table 1, dogs 1 and 2). Differences between high and low velocities indicate a stenotic flow pattern and might allow identification of locations of stenosis in dogs. Similar to the previous study in dogs, ^29^ peak velocities were lower in all dorsal SAS compared to the ventral SAS. A reduced peak velocity within the dorsal SAS at the level of the FM was observed in CKCS compared to the control group, irrespective of signs of syringomyelia or Chiari‐like malformation.^29^ It is unclear if such differences in flow velocity are due to morphological abnormalities present in CKCS, and further comparison with other dog populations will shed further light on this question.

The diagnostic value of PC‐MRI in dogs needs to be investigated in future studies. Currently, it is unclear if the method will help to identify sites of stenosis or explain other alterations of CSF spaces. Further future applications of CSF flow measurements are the assessment of the intracranial compliance.^15^, ^43^, ^44^, ^45^ As a first step, it was important to develop an examination protocol suitable for the clinical setting and to have velocity data available from neurologically healthy dogs as a baseline for further comparisons. Possibly, single sequence parameters might need to be adapted for different scanner types, but especially the information about velocity encoding and the resulting flow velocities are of value for future applications. In conclusion, we provide a 2D PC‐MRI examination protocol with measurements at 3 different locations that seems suitable for the clinical setting. The acquired CSF flow from 2D PC‐MRI of healthy dogs provides normative values for future applications in research and clinical tests.

## CONFLICT OF INTEREST DECLARATION

Authors declare no conflict of interest.

## OFF‐LABEL ANTIMICROBIAL DECLARATION

Authors declare no off‐label use of antimicrobials.

## INSTITUTIONAL ANIMAL CARE AND USE COMMITTEE (IACUC) OR OTHER APPROVAL DECLARATION

Approved by the Cantonal Veterinary Office of Zurich according to the Swiss national animal protection law with the animal permission number ZH2016/17.

## HUMAN ETHICS APPROVAL DECLARATION

Authors declare human ethics approval was not needed for this study.
